# Application of DMAIC Cycle and Modeling as Tools for Health Technology Assessment in a University Hospital

**DOI:** 10.1155/2021/8826048

**Published:** 2021-08-17

**Authors:** Alfonso Maria Ponsiglione, Carlo Ricciardi, Arianna Scala, Antonella Fiorillo, Alfonso Sorrentino, Maria Triassi, Giovanni Dell'Aversana Orabona, Giovanni Improta

**Affiliations:** ^1^Department of Electrical Engineering and Information Technology (DIETI), University of Naples “Federico II”, Naples, Italy; ^2^Department of Advanced Biomedical Sciences, University of Naples “Federico II”, Naples, Italy; ^3^Department of Public Health, University Hospital of Naples “Federico II”, Naples, Italy; ^4^Maxillofacial Surgery Unit, Department of Neurosciences, Reproductive and Odontostomatological Sciences, University Hospital of Naples “Federico II”, Naples, Italy

## Abstract

**Background:**

The Health Technology Assessment (HTA) is used to evaluate health services, manage healthcare processes more efficiently, and compare medical technologies. The aim of this paper is to carry out an HTA study that compares two pharmacological therapies and provides the clinicians with two models to predict the length of hospital stay (LOS) of patients undergoing oral cavity cancer surgery on the bone tissue.

**Methods:**

The six Sigma method was used as a tool of HTA; it is a technique of quality management and process improvement that combines the use of statistics with a five-step procedure: “Define, Measure, Analyze, Improve, Control” referred to in the acronym DMAIC. Subsequently, multiple linear regression has been used to create two models. Two groups of patients were analyzed: 45 were treated with ceftriaxone while 48 were treated with the combination of cefazolin and clindamycin.

**Results:**

A reduction of the overall mean LOS of patients undergoing oral cavity cancer surgery on bone was observed of 40.9% in the group treated with ceftriaxone. Its reduction was observed in all the variables of the ceftriaxone group. The best results are obtained in younger patients (−54.1%) and in patients with low oral hygiene (−52.4%) treated. The regression results showed that the best LOS predictors for cefazolin/clindamycin are ASA score and flap while for ceftriaxone, in addition to these two, oral hygiene and lymphadenectomy are the best predictors. In addition, the adjusted *R* squared showed that the variables considered explain most of the variance of LOS.

**Conclusion:**

SS methodology, used as an HTA tool, allowed us to understand the performance of the antibiotics and provided variables that mostly influence postoperative LOS. The obtained models can improve the outcome of patients, reducing the postoperative LOS and the relative costs, consequently increasing patient safety, and improving the quality of care provided.

## 1. Introduction

Healthcare seeks to give improvements in the prevention, control, and treatment of diseases, but at the same time, it also deals with complications, inefficiencies, and other problems that put patients' safety at risk. Therefore, it is necessary to monitor the health services provided by applying management methods and tools to control quality [1]. Nowadays, several methodologies and approaches are used in healthcare to help in the clinical decision-making process [2–8], to aid physicians in defining the diagnosis and prognosis of patients [9–11], and to analyze quality improvement in hospital processes [12, 13]. A useful methodology for these purposes is the Health Technology Assessment (HTA), a multidisciplinary process for medical-clinical, social, organizational, economic, technological, ethical, and legal implication analysis of health technology through the evaluation of efficiency, security, costs, and social and organizational impact [14, 15]. The technologies could be drugs, medical devices, vaccines, procedures, and, generally, all systems developed to solve a health problem and to improve the quality of life.

Parmar and Chan [16] used HTA methodology in urologic oncology. As a result of the rapid development of new cancer therapies, it is important to have a decision-making tool which leads to the choice of the right therapy in a short period of time. In this study, HTA was used as an approach that could help to guide value-based decision-making. An HTA model was developed for the evaluation of generic pharmaceutical products. This tool allows us to compare, both qualitatively and economically, equivalent drug preparation. HTA was employed to evaluate a new health technology for the thyroglobulin assay in patients with differentiated thyroid cancer. The authors used the Dynamic AHP as an HTA tool to reach the goal [17]; this paper proved also the utility of combining HTA with other managerial approaches.

Another promising tool to improve the quality of healthcare processes is Six Sigma (SS) [18–21]. Initially introduced in the manufacturing sector, today, it is widely developed in the health sector. SS relies on the “Define, Measure, Analyze, Improve, Control” cycle (DMAIC), which is a five-step procedure related to quality management and process improvement that exploits both statistical and managerial tools. Through this problem-solving strategy with a fixed structure, it is possible to analyze a process in order to improve its performance reducing the “natural variability” and carry out the “systematic control” of the critical variables to obtain a better result. The procedure is divided into the following phases: defining the project goals and customer (internal and external) requirements, measuring the process to determine current performance, analyzing and defining the root cause(s) of relevant defects, improving the process by eliminating defect root causes, and controlling future process performance. For the first time, Bill Smith developed this methodology in 1986 with the aim of reducing product or process defects that did not satisfy customers [18, 22]. DMAIC is then a framework used to enable the team to define and achieve set objectives [1, 23, 24].

From literature studies, it stands out the success that the strength of SS is founded not only in the manufacturing field but also in the health sector, where the SS DMAIC approach has been applied, for example, to improve first aid processes [25] and in the paramedical services [26]. Mahesh et al. [27] demonstrated how to reduce patients' waiting time to receive a specialist medical visit at the Out-Patient Department of Cardiology in a private hospital in the city of Bangalore, and El-Eid et al. [28] have confirmed SS as an efficient and effective management tool to improve the patient discharge process, reducing patient discharge time. As well, other studies confirmed the validity of the methodology [13, 29–33], also in combination with other methods such as the Agile [34]. Ricciardi et al. [12] analyzed the introduction of the Diagnostic Therapeutic Assistance Path (DTAP), employing Lean Thinking and SS methodology based on the DMAIC cycle. Furthermore, several studies show that the SS is often associated with Lean Thinking: this approach aims to improve services to meet customer needs by eliminating wastes and reducing costs [35–37]. The use of these methodologies has reported multiple benefits in healthcare; in fact, they have been used to improve clinical decision-making processes and to reduce the risk of healthcare-associated infections in surgery departments [38], while others have conducted studies to introduce prehospitalization to perform the necessary tests and examinations for hip and knee prosthetic surgery [29, 39].

The problem of healthcare infections is of great interest in many surgery departments, and it is an indicator of hospital efficiency, safety, and quality. Scotton et al. [40] conducted a study whose purpose was to analyze infections in patients after Salvage Laryngectomy (SL) and review the potential impact of the antibiotic prophylaxis adopted. The results showed that infection rates after SL were high, and univariate analysis demonstrated risk variables that had a significant correlation with infection, so the antibiotic regimen is probably ineffective. Other authors [41–48] presented an overview of current evidence-based best practices in the use of prophylactic antibiotics in head and neck cancer surgery; indeed, this type of patient is at high risk of developing complications after surgery. Thus, they reported that prophylactic antibiotics helped significantly reduce the risk of infection [49]. However, short four-dose antibiotic regimens for 24 hours are as effective as prolonged cycles, regardless of the complexity of the procedure [50–53]. In the same framework, the research of Egan et al. [54] discusses the use of the SS focusing on therapy with antimicrobial gentamicin, which requires good practice in selecting the dose and monitoring serum levels. They found a new dosage with a standardized sampling, a monitoring program, and a new timing of drug delivery that maximized local capacities. In light of the above-mentioned studies, it emerges the importance of choosing correct prophylactic antibiotics to manage patients appropriately after surgical interventions.

To this aim, in our recent study [55], SS was employed to compare the use of antibiotics in patients undergoing oral cancer surgery on bone tissue. Starting from the previous promising results, in this work, two antibiotics, ceftriaxone and the combination of cefazolin and clindamycin, are compared in order to understand which one reduces the postoperative length of hospital stay (LOS) for patients undergoing oral cavity cancer surgery on the bone tissue. In this study, it is taken into consideration the clinical factor because the two antibiotics are quite similar from a safety, legal, ethical, economic, and technological point of view. Six Sigma (SS) methodology is applied as a tool of HTA in order to achieve the aim. SS was used to analyze the influence of some clinical variables (ASA score, age, gender, oral hygiene, diabetes, and cardiovascular diseases) on the Critical to Quality (CTQ) (postoperative LOS). Patients' postoperative LOS can be described as the duration of time after a patient's surgery until the day of discharge.

The novelty of this new study is the use of the DMAIC cycle as an HTA tool including a modeling phase. This would enable healthcare providers to understand the performance of antibiotics, improving patients' outcomes, reducing postoperative LOS and related costs, consequently, increasing patient safety, and improving the quality of care provided. After applying DMAIC, a modeling study was conducted through a multinomial linear regression; in particular, it was applied to obtain two models capable of predicting postoperative LOS for each antibiotic. In order to do this, we included the surgical variables that were considered in the previous study [55].

## 2. Materials and Statistical Tools

SS and subsequently the modeling phase were used to implement the HTA methodology. In detail, deploying the DMAIC cycle, characteristic of SS, means developing five phases:

1. The Define phase identifies the customers and the objectives to be reached will be established [27] allowing a team to identify the problem
2. The Measure phase defines the main characteristics of the process and the parameters that will lead to improvement [56]
3. The Analyze phase is used to understand the influence of the collected variables on the CTQ or to evaluate the data collected in the previous phases of the study using various analytical tools available such as regression analysis, fishbone diagram, tree diagrams, and brainstorming
4. The Improve phase employs all the previous analyses to design changes in a process and to improve the performance, i.e., introducing a new antibiotic protocol
5. The Control phase is employed to monitor the whole process and, in this research, to compare the performance of the drugs

SS led the way for the development of the modeling phase, providing us with information about all the variables. Modeling allowed us to enrich the univariate analysis with a multivariate one and to implement a tool able to predict the postoperative LOS for each patient. These models will be very useful for both ward management and hospital management. Predicting the LOS of a patient determines a more efficient hospital bed organization, a better management of nurses and doctors on duty, and lastly, a cost reduction for hospitals. Thus, combining SS and modeling could be considered a valuable tool for HTA methodology.

In conclusion, the purpose of this paper is to assess the performance of two antibiotics, cefazolin plus clindamycin [57, 58] and ceftriaxone [59], through an HTA by using SS and modeling as a tool in the framework of oral cavity cancer surgery on bone tissues.

### 2.1. The Clinical Case Study

In this study, two groups of patients with oral cancer starting from the bone were analyzed: the first one was treated with ceftriaxone between 2006 and 2011, while the second one was treated with cefazolin and clindamycin between 2011 and 2019. The cefazolin group consisted of 54 patients, while the other by 51 patients. Oral cancer is the sixth most common cancer in the world [60] but the ones starting from the jaws are rare. The majority of the oral cancers affecting the bone derives from the epithelial quote of the oral mucosa, but there are also cancers that originally start from the bones, which are rare. Sarcomas are very rare tumors in the head and neck district, osteosarcoma being the most common of them [61]. They represent 1% of all the malignancies affecting the head and neck [62]. The incidence of sarcomas starting from the mandibles ranges from 4% to 10% [63]. In this study, we decided to analyze also those patients affected by ameloblastomas, which is not actually a malignant neoplasm. This choice is due to the fact that in the case of big ameloblastomas affecting the jaws, a big removal of tissue and reconstruction with the same surgical techniques used for patients affected by oral bone cancers are often required. The data was taken from printed medical records. Statistical tests, useful for analyses, were carried out with IBM SPSS.

For the collection of data, some inclusion and exclusion criteria were taken into consideration:

- All patients were included without exclusion due to medical history (gender, age, cardiovascular diseases, diabetes, oral hygiene, American Society of Anaesthesiologists (ASA) Score)
- Patients with cancers starting from the bones or starting from the oral mucosa and then affecting the bone were included. We also included patients with ameloblastomas because of their osteolytic patterns
- Patients treated in “day surgery” were excluded
- Patients with too many missing data were not included because they would compromise the analysis
- Patients with a change of the antibiotic therapy during their recovery, because no evidence of efficacy, were not included in the analysis, but their number was recorded as it is a qualitative indicator of treatment failure
- Patients allergic to cefazolin and clindamycin or ceftriaxone were excluded

As regards the Unit of Maxillofacial Surgery, the ward consists of 9 rooms with 22 beds for the patients and some more rooms for surgeons and nurses. The Operatory Block of the Department disposes of two operating rooms.

Oncological maxillofacial surgery is a branch of maxillofacial surgery which deals with the surgical approach to head and neck malignancies and the reconstruction of the lost tissues [64].

When no allergy was described, from 2006 to 2011, a postoperative antibiotic protocol with ceftriaxone was used. Since 2011, there has been a shift to the use of the association of cefazolin plus clindamycin as postoperative antibiotic prophylaxis.

### 2.2. The Development of the Six Sigma: The Define Phase

The purpose of the “Define” phase is to define a multidisciplinary workgroup and to divide the tasks for the analysis. The team consists of clinicians from the Maxillofacial Department of the University Hospital “Federico II” of Naples, an economist, and biomedical engineers with experience in health management. The team was responsible for collecting and analyzing data of patients with oral cavity cancer considering the influence of some variables. The sample and the leader supervised and coordinated the study and interpretation of the data. A project diagram was created to define the problem to be solved:

(i) *Project Title*. Health Technology Assessment between two antibiotics in the context of Maxillofacial Surgery
(ii) *Question*. Investigation of the best antibiotics in the analyzed context
(iii) *Critical to Quality*. Postoperative LOS
(iv) *Target*. Realize corrective measures to reduce the CTQs
(v) *Deliverables*. The performance of cefazolin/clindamycin and ceftriaxone, the outcome of patients, reducing postoperative LOS, and the related costs
(vi) Timeline:
  1. Define: January 2010
  2. Measure: January 2010
  3. Analyze: January 2010
  4. Improve: January 2011
  5. Control: 2011–2018
(vii) *In Scope*. Oral cavity cancer surgery on bone tissues. Maxillofacial surgery in the University Hospital of Naples “Federico II”
(viii) *Out of Scope*. All the other structures and interventions and drugs
(ix) *Financial*. No funding to reach the target
(x) *Business Need*. Identifying the best antibiotic for the surgery under examination

### 2.3. Dataset Description: The Measure Phase

The data collected from the medical records at the Department of Maxillofacial Surgery were selected according to the inclusion and exclusion criteria. After applying the inclusion and exclusion criteria, the first sample of data concerned patients treated with ceftriaxone from 2006 to 2011 (45 patients), and the other sample of data (48 patients) was referred to patients treated with cefazolin and clindamycin from 2011 to 2019. The variables used to compare the two antibiotics were

- Gender
- Age
- American Society of Anaesthesiologists (ASA) Score
- Quality of oral hygiene
- Diabetes
- Cardiovascular diseases

Other variables were analyzed through univariate analysis in a previous study [55]; thus, they were included only in the modeling phase. Descriptive characteristics of the dataset were carried out for the postoperative LOS variables: the results for cefazolin/clindamycin were, respectively, an average of 16.51 days and a variance of 62.21. Instead, the results for ceftriaxone were an average of 9.75 days and a variance of 66.81.

**Figure 1:**
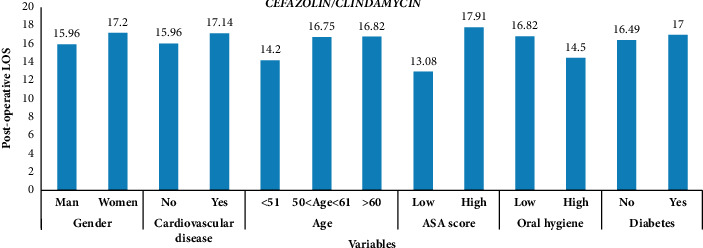
Mean postoperative LOS for each mode of variables regarding cefazolin/clindamycin.

We drew a histogram (Figure 1) showing the mean postoperative LOS of patients, measured in days, submitted to the administration of cefazolin/clindamycin according to each variable. The highest average LOS is for patients with a high ASA score, while the lowest is for patients with a low ASA score.

**Figure 2:**
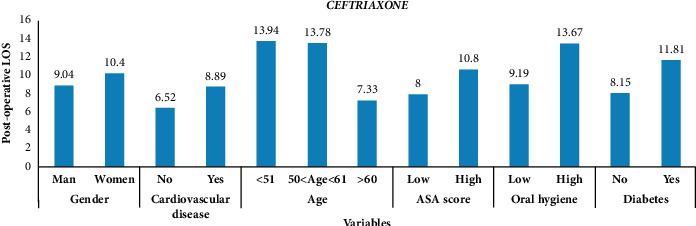
Mean postoperative LOS for each mode of variables regarding ceftriaxone.

Figure 2 shows the distribution of mean postoperative LOS of patients who used ceftriaxone. Patients below the age of 51 have the highest mean LOS, whereas those without cardiovascular disease have the lowest mean LOS.

### 2.4. Statistical Analysis: The Analyze Phase

**Figure 3:**
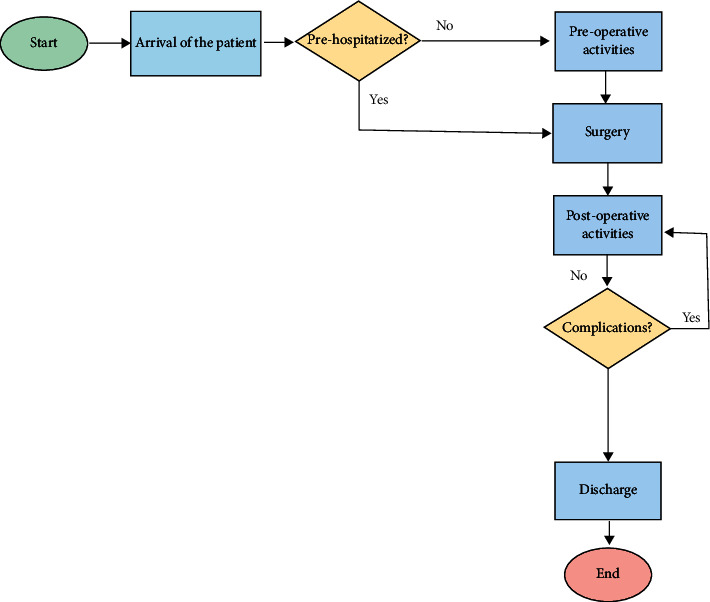
The flowchart of the hospitalization process for patients undergoing oncologic surgery at the Maxillofacial Department of the University Hospital of Naples “Federico II.”

In Figure 3, patients' pathway is shown from the arrival at the hospital to the discharge. They arrived at the hospital; then, if they receive a previous prehospitalization, they undergo surgery directly; otherwise, they are subjected to preoperative activities before surgery. Finally, if there are complications after the surgery, the patient undergoes postoperative activities; otherwise, they will be discharged after fewer days.

A Kolmogorov–Smirnov test showed a *p* value lower than 0.0001. In order to understand the variables that could influence the postoperative LOS in the ceftriaxone group, nonparametric tests were employed: Mann–Whitney and Kruskal–Wallis (only for age). In this case, some significant *p* values were found for age and ASA score while the *p* value of cardiovascular disease was almost significant (*p* value = 0.066) (Table 1).

**Figure 4:**
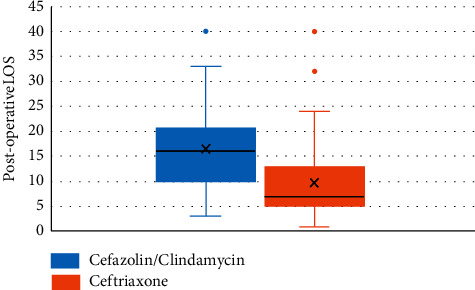
Boxplot of the mean postoperative LOS for “cefazolin/clindamycin” and “ceftriaxone” groups.

A box diagram was developed and is shown in Figure 4, which clearly highlights the decrease in the ceftriaxone group of LOS, measured in days.

The Control phase allowed us to monitor and guarantee the sustainability of the long-term continuous improvement of the performance. Thus, the team identified the following actions:

- Periodic review meetings to evaluate the maxillofacial surgery process
- Internal audit to verify the performance of antibiotics
- Production of reports that highlight the trend of patients' postoperative patients measured in days

After analyzing the data according to the DMAIC cycle, the modeling phase started by implementing the multiple linear regression. It is also known simply as multiple regression and is a statistical technique that uses several explanatory variables to predict the outcome of a response variable. The goal of multiple linear regression is to model the linear relationship between the explanatory (independent) variables and response (dependent) variables. In other words, multiple regression is the extension of ordinary least-squares (OLS) regression that involves more than one explanatory variable.

In this study, it was used to obtain a model capable of predicting the postoperative LOS for each patient undergoing oral cavity cancer surgery on the bone. In order to obtain the best models, we considered also the surgical variables that were studied in a previous research on the same topic [55]. Therefore, the considered variables in order to implement the model were 11: gender, age, ASA score, the quality of oral hygiene, diabetes, cardiovascular diseases, tracheotomy, lymphadenectomy, infections, dehiscence, and flap.

## 3. Results

### 3.1. Statistical Analysis for Cefazolin plus Clindamycin

The Kolmogorov–Smirnov test was applied to investigate the distribution of the postoperative LOS data regarding cefazolin/clindamycin; a *p* value of 0.200 indicated a normality distribution. Thus, to investigate the variables potentially influencing postoperative LOS, *t*-test and ANOVA were employed. The results are represented in Table 2. No significance was found in the tests, but the difference between postoperative LOS in each category gave insights about a potential influence in many of the variables; the ASA score was almost significant.

### 3.2. Comparison between the Two Antibiotics: The Control Phase

The Kolmogorov–Smirnov test showed a *p* value of less than 0.0001; i.e., the data were not normally distributed. The results of the comparison between the two antibiotics through Mann–Whitney and Kruskal–Wallis tests with an alpha level of 0.05 are shown in Table 3. Overall, the difference in postoperative LOS between the cefazolin/clindamycin and ceftriaxone groups was statistically significant with a reduction of 40.9%. All tests were statistically significant among the mode of variables, except for older patients (>60 years with a *p* value of 0.117). The greatest reduction in postoperative LOS results in younger patients (<51 years with a reduction of 54.1%) and people with low oral hygiene (52.4%).

Table 4 shows the results of a study regarding the frequencies of each variable, obtained by performing a chi-square test. A statistically significant difference between the occurrences of cefazolin/clindamycin and ceftriaxone groups was obtained according to age, ASA score, and oral hygiene.

### 3.3. Combining SS and Modeling

The statistical analysis was useful for the subsequent modeling phase. As mentioned in the introduction, in this phase, we also considered some surgical variables analyzed in a preceding paper [55]. For both antibiotic protocols, the multiple linear regression was implemented obtaining two predictive models whose equations are shown as follows:

(1) $$\begin{array}{c} y_{1}=\beta_{1}x_{1}+\beta_{2}x_{2}+\beta_{3}x_{3}+\beta_{4}x_{4}+\;\beta_{5}x_{5}+\beta_{6}x_{6}+\beta_{7}x_{7}+\beta_{8}x_{8}+\varepsilon_{1}, \end{array}$$

(2) $$\begin{array}{c} y_{2}=\partial_{1}x_{1}+\partial_{2}x_{2}+\partial_{3}x_{3}+\partial_{4}x_{4}+\;\partial_{5}x_{5}+\partial_{6}x_{6}+\varepsilon_{2}, \end{array}$$

where *y*_1_ represents the LOS of patients treated with cefazolin/clindamycin, *y*_2_ the LOS of patients treated with ceftriaxone, *x*_*i*_ the considered variables, *β*_*i*_ and ∂_*i*_ the regression coefficients, and *ε*_*i*_ the errors.

Before carrying out the regression analysis, it is necessary to verify, for both antibiotics, the hypotheses given in Table 5 which also contains references to the additional material provided in order to give more details on these verifications.

As shown in equations (1) and (2), not all variables were considered for both models. In particular, 8 variables were included for cefazolin/clindamycin (ASA score, diabetes, cardiovascular disease, tracheotomy, lymphadenectomy, infections, dehiscence, and flap) while 6 variables were included for ceftriaxone (ASA score, oral hygiene, diabetes, cardiovascular disease, lymphadenectomy, and flap). The exclusion criteria of variables in each model were as follows:

- Gender and age were excluded in order to obtain models based on clinical factors
- Oral hygiene was excluded from the cefazolin/clindamycin model because it did not respect the “absence of multicollinearity” hypothesis; i.e., there was a dependency between it and the ASA score variable. Since ASA score had a lower *p* value in the previous analyses of DMAIC than oral hygiene, the latter was excluded
- Infections and dehiscence were excluded from the ceftriaxone model because no patient has experienced them. Similarly, the tracheotomy variable was excluded because there was only one case and it was not enough

Tables 6 and 7 show the regression coefficients, errors, and statistical significance obtained for each variable.

The results show that for cefazolin/clindamycin the ASA score is statistically significant and the flap is very close to the *p* value of 0.05. Similarly, for ceftriaxone the ASA score and the flap are variables that have a significant effect on LOS, as well as oral hygiene and lymphadenectomy.

A summary of the two models is given in Table 8. In particular, there are the coefficient of determination (*R*^2^), the adjusted R squared, and the standard error of the estimate.

Since the two models have a different number of predictors, in addition to the *R*^2^, the adjusted *R* squared has also been reported; it is a modified version of *R*^2^, adjusted according to the number of predictors in the model. Although there are also other variables affecting LOS, the results obtained indicate that, for both antibiotics, about 82–89 percent of the variance in LOS is explained by the selected variables.

## 4. Discussion and Conclusion

Over the past few years, the healthcare sector has paid attention to cost increases, mainly due to the drop of refunds, and to improve the experience of patients. In this scenario, the HTA provides health leaders with a useful tool to improve the efficiency and effectiveness of clinical processes; this tool has become fundamental in healthcare due to the high amount of medical device patents that have been required in the last decades [65]. In the literature, some studies applied the HTA to support decision-making processes regarding the purchase of medical devices [66] or drug refund policies [67, 68], while only a few works present an application of the HTA for evaluating the introduction of new antibiotic prophylaxis. In this study, we tackled this issue by employing a combination of both SS and HTA. In particular, encouraged by the results achieved in previously published studies [7, 55], here we adapted the framework of the SS DMAIC cycle to build a tool that could support the HTA of a new antibiotic prophylaxis procedure for patients undergoing oral cancer surgery of the bone. The assessment has been made taking into account a healthcare key performance indicator, which is the postoperative LOS. Indeed, the LOS is a useful metric to determine the economic, organizational, and clinical impact of healthcare services. In this work, a multiple regression model has been integrated within the SS framework to investigate the relationships between a prolonged LOS and the prophylaxis procedure in order to determine the impact of the introduction of a new antibiotic on the hospital stay. When framed into the Improve phase of the SS DMAIC cycle, the regression model helped in determining the effect of the new antibiotic prophylaxis on the postoperative LOS and enabled a comparison between the two antibiotics, thus providing an additional informative tool to support the decision-making process, in accordance with our previous works [7, 55]. The results obtained from the comparative statistical analysis (Table 3) showed a 41% reduction in the LOS for patients treated with ceftriaxone compared to those treated with cefazolin/clindamycin, with the highest decrease achieved among younger patients (−54.1%). This could be due to the better response of younger patients toward the performed surgical procedure, as opposed to older patients, whose surgical intervention can be influenced by possible comorbidities and other variables, in accordance with the literature [69, 70]. The modeling phase with the two regression models (Tables 6 and 7) enabled the identification of the variables, among demographic, clinical, and surgical ones as considered in a previous study [55], which influence the postoperative LOS the most and provided promising tools for the prediction of the LOS in patients undergoing oral cavity cancer surgery on the bone who are treated with cefazolin/clindamycin or with ceftriaxone. Of note, during the whole study's range of time, the choice of the antibiotics was completely independent of the research. Indeed, the antibiotic to be administered was defined by the hospital's protocols which change the antibiotic choice in 2011 according to the new trends of therapy described in the medical literature.

In summary, the proposed approach confirmed the value of combining both the SS DMAIC approach and modeling, which can serve as a tool to support HTA processes for understanding the optimal therapeutic approach.

In conclusion, this HTA study confirmed and further extended the results achieved and presented in the literature which considered the ceftriaxone as the best option for patients undergoing oral cancer surgery on bone tissue [55] and provides the health policy with two important results: the antibiotic which reduces the postoperative LOS and two models which predict it. Succeeding in predicting the postoperative LOS of a patient could lead to many benefits for both the hospital and patients. Indeed, the hospital could better manage all its resources, reduce waste and costs, and improve the understanding of patients' needs, which are all aims of an SS project; meanwhile, the patients could experience a better quality of care and a lower LOS.

The evaluation of antibiotic performance is an important topic, as it is linked to healthcare‐associated infections in hospitals, as evidenced by studies in the literature. This paper evaluates the performance of antibiotics considering the most important variables in the maxillofacial area. In addition, the DMAIC approach implies a positive advantage, giving support to the medical staff in the decision-making process of antibiotic administration, reducing the gap between practice and theory. Therefore, the reduction of postoperative LOS and the rate of infections of patients undergoing oral cavity cancer surgery benefit both the hospital and patients: patients satisfied in terms of a few days of hospitalization and effective and efficient therapy, while the hospital has more available beds and saves costs of managing patients with complications.

## Figures and Tables

**Table 1:** The analysis of potential factors influencing postoperative LOS for the “ceftriaxone” group.

Variable	Category	*N*	LOS (mean ± std. dev.)	*p* value
Gender	Men	25	9.04 ± 7.49	0.669
	Women	23	10.40 ± 9.02	

Age	<51	21	6.52 ± 5.33	**0.013** ^*∗*^
	50 < age < 61	9	8.89 ± 6.92	
	>60	18	13.94 ± 10.04	

ASA score	Low	30	7.33 ± 5.84	**0.007**
	High	18	13.78 ± 10.15	

Oral hygiene	Low	30	8.00 ± 6.74	0.306
	High	18	10.80 ± 9.00	

Diabetes	No	42	9.19 ± 8.05	0.213
	Yes	6	13.67 ± 9.46	

Cardiovascular disease	No	27	8.15 ± 7.48	0.066
	Yes	21	11.81 ± 8.92	

^*∗*^Kruskal–Wallis test.

**Table 2:** The analysis of potential factors influencing postoperative LOS for the “cefazolin/clindamycin” group.

Variable	Category	*N*	LOS (mean ± std. dev.)	*p* value
Gender	Men	25	15.96 ± 7.32	0.606
	Women	20	17.20 ± 8.68	

Age	<51	5	14.20 ± 7.26	0.793^*∗*^
	50 < age < 61	12	16.75 ± 9.11	
	>60	28	16.82 ± 7.65	

ASA score	Low	13	13.08 ± 6.69	0.062
	High	32	17.91 ± 8.00	

Oral hygiene	Low	39	16.82 ± 8.17	0.509
	High	6	14.50 ± 5.89	

Diabetes	No	43	16.49 ± 8.07	0.930
	Yes	2	17.00 ± 0.00	

Cardiovascular disease	No	24	15.96 ± 8.65	0.621
	Yes	21	17.14 ± 7.07	

^*∗*^ANOVA test.

**Table 3:** The complete comparative statistical analysis. Mann–Whitney and Kruskal–Wallis were used, respectively, for dichotomous groups and for the age group.

Variable	Category	Cefazolin/clindamycin (mean ± std. dev.)	Ceftriaxone (mean ± std. dev.)	Difference (%)	*p* value
All patients		16.51 ± 7.89	9.75 ± 8.26	−40.9	**<0.0001**

Gender	Men	15.96 ± 7.32	9.04 ± 7.49	−43.4	**0.003**
	Women	17.20 ± 8.68	10.40 ± 9.02	−39.5	**0.002**

Age	<51	14.20 ± 7.26	6.52 ± 5.33	−54.1	**0.015** ^*∗*^
	50 < age < 61	16.75 ± 9.11	8.89 ± 6.92	−46.9	**0.028** ^*∗*^
	>60	16.82 ± 7.65	13.94 ± 10.04	−17.4	0.117^*∗*^

ASA score	Low	13.08 ± 6.69	7.33 ± 5.84	−44.0	**0.007**
	High	17.91 ± 8.00	13.78 ± 10.15	−23.1	**0.042**

Oral hygiene	Low	16.82 ± 8.17	8.00 ± 6.74	−52.4	**0.001**
	High	14.50 ± 5.89	10.80 ± 9.00	−25.5	**0.040**

Diabetes	No	16.49 ± 8.07	9.19 ± 8.05	−44.2	**<0.0001**
	Yes	17.00 ± 0.00	13.67 ± 9.46	n.a.	n.a.

Cardiovascular disease	No	15.96 ± 8.65	8.15 ± 7.48	−48.9	**<0.0001**
	Yes	17.14 ± 7.07	11.81 ± 8.92	−31.2	**0.012**

^*∗*^Kruskal–Wallis test; n.a.: not applicable.

**Table 4:** The analysis of the frequencies for each variable is performed through a chi-square test.

Variable	Category	Cefazolin/clindamycin (*N*)	Ceftriaxone (*N*)	*p* value
Gender	Men	25	25	0.737
	Women	20	23	

Age	<51	5	21	0.002
	50 < age < 61	12	9	
	>60	28	18	

ASA score	Low	13	30	0.001
	High	32	18	

Oral hygiene	Low	39	30	0.008
	High	6	18	

Diabetes	No	43	42	0.166
	Yes	2	6	

Cardiovascular disease	No	24	27	0.778
	Yes	21	21	

**Table 5:** Verification of the assumptions of multiple regression models for both antibiotics and reference to corresponding Supplementary Material items.

Assumption	Description	Reference to Supplementary Material
Linearity	Verify if a linear relationship exists between the dependent variable and each predictor of the model	Figures S1 and S2
Independence of residuals	Verify if the errors of the model are independent	Tables S1
Collinearity	Verify if the predictors are not linearly correlated with each other	Table S2
Outliers	Verify if there are influential cases biasing the model	Figure S3
Normality of the residuals	Verify if the errors of the model are normally distributed	Figure S4
Homoscedasticity	Verify if the variance of the errors of the model is constant	Figure S5

**Table 6:** Regression coefficients, errors, and *p* value for cefazolin/clindamycin model.

Variables	Unstandardized regression coefficients (cefazolin/clindamycin)		
	Regression coefficients (*β*_*i*_)	Std. error	*p* value
ASA score	3.406	0.506	**0.000**
Diabetes	1.025	4.066	0.803
Cardiovascular disease	2.541	1.707	0.147
Tracheotomy	0.022	2.366	0.993
Lymphadenectomy	2.816	2.139	0.198
Infections	2.790	3.383	0.416
Dehiscence	2.636	2.507	0.301
Flap	3.617	1.824	0.056

**Table 7:** Regression coefficients, errors, and *p* value for ceftriaxone model.

Variables	Unstandardized regression coefficients (ceftriaxone)		
	Regression coefficients (*δ*_*i*_)	Std. error	*p* value
ASA score	2.272	0.609	**0.001**
Oral hygiene	0.873	0.358	**0.020**
Diabetes	4.938	2.546	0.600
Cardiovascular disease	-0.423	1.732	0.808
Lymphadenectomy	14.174	5.592	**0.015**
Flap	6.991	2.340	**0.005**

**Table 8:** Coefficient of determination, adjusted *R* squared, and standard errors of the two models.

	Cefazolin/clindamycin	Ceftriaxone
*R* ^2^	0.914	0.847
Adjusted *R* squared	0.892	0.823
Std. error	5.218	4.799

## Data Availability

Data are not present in a publicly accessible repository. Data could be made available upon reasonable request to the authors.

## Abbreviations

ASA: American Society of Anaesthesiologists
CTQ: Critical to quality
DMAIC: Define, Measure, Analyze, Improve, Control
LOS: Length of hospital stay
SPSS: Statistical Software for the Social Sciences
SS: Six Sigma.

## Supplementary Materials

Supplementary Materials Details on the multiple regression model assumptions check (as briefly summarized in Table 5 of the manuscript) are reported in the attached Supplementary Material file.
