# Supplementary material for: Application of DMAIC Cycle and Modeling as Tools for Health Technology Assessment in a University Hospital
Source: J Healthc Eng. 2021 Aug 17;2021:8826048. doi: 10.1155/2021/8826048 (PMC8387173; doi:10.1155/2021/8826048)
Supplement: Supplementary Materials — Details on the multiple regression model assumptions check (as briefly summarized in Table 5 of the manuscript) are reported in the attached Supplementary Material file. [file 8826048.f1.docx]

# SUPPLEMENTARY MATERIAL

**TITLE**

Application of DMAIC cycle and modelling as tools for Health Technology Assessment in a university hospital.

**AUTHORS**

Alfonso Maria Ponsiglione^1,§^, Carlo Ricciardi^2,§^, Arianna Scala^3,^*, Antonella Fiorillo^2^, Alfonso Sorrentino^4^, Maria Triassi^3^, Giovanni Dell’Aversana Orabona^4,‡^ and Giovanni Improta^3,‡^

**AFFILIATIONS**

^1^ Department of Electrical Engineering and Information Technology (DIETI), University of Naples “Federico II”, Naples, Italy.

^2^ Department of Advanced Biomedical Sciences, University of Naples “Federico II”, Naples, Italy.

^3^ Department of Public Health, University Hospital of Naples “Federico II”, Naples, Italy.

^4^ Maxillofacial Surgery Unit, Department of Neurosciences, Reproductive and Odontostomatological Sciences, University Hospital of Naples “Federico II”, Naples, Italy.

^§^ These authors contributed equally to this work

^‡^ These authors contributed equally to this work

* corresponding author

**CORRESPONDING AUTHOR INFORMATION**

Arianna Scala

University of Naples “Federico II”

Department of Public Health

mobile +393476790092

Email: ariannascala7@gmail.com

# 1. MULTIPLE REGRESSION MODEL

Prior to estimating the final values of the coefficient of the Multiple Regression model, the following assumptions are checked for both antibiotics:

- Linearity: to verify if a linear relationship exist between the dependent variable and each predictors of the model;
- Independence of the residuals: to verify if the errors of the model are independent;
- Collinearity: to verify if the predictors are not linearly correlated with each other’s;
- Outliers: to verify if there are influential cases biasing the model;
- Normality of the residuals: to verify if the errors of the model are normally distributed;
- Homoscedasticity: to verify if the variance of the errors of the model is constant.

### **1.1 LINEARITY**

Partial regression plots are used to identify the linear relationship between the Length of Stay (LOS) and selected predictor. Partial regression plots show on the x-axis the residuals of the independent variable, i.e. the errors obtained in modelling a given variable using all the other predictors, and on the y-axis the residuals of the LOS modelled excluding that specific independent variable. The plots for the two antibiotics are displayed in Figures S1 and S2.


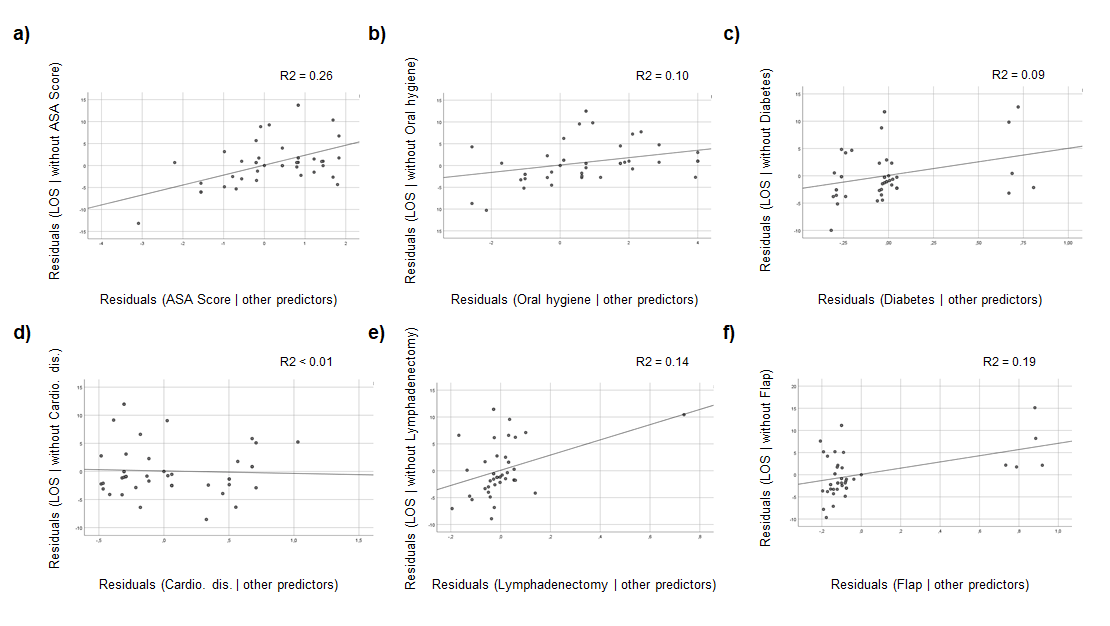


**Figure S1.** Partial regression plots for Cefriaxone displayed on each predictor: **a)** ASA Score; **b)** Oral hygiene; **c)** Diabetes; **d)** Cardiovascular disease; **e)** Lymphadenectomy; **f)** Flap.


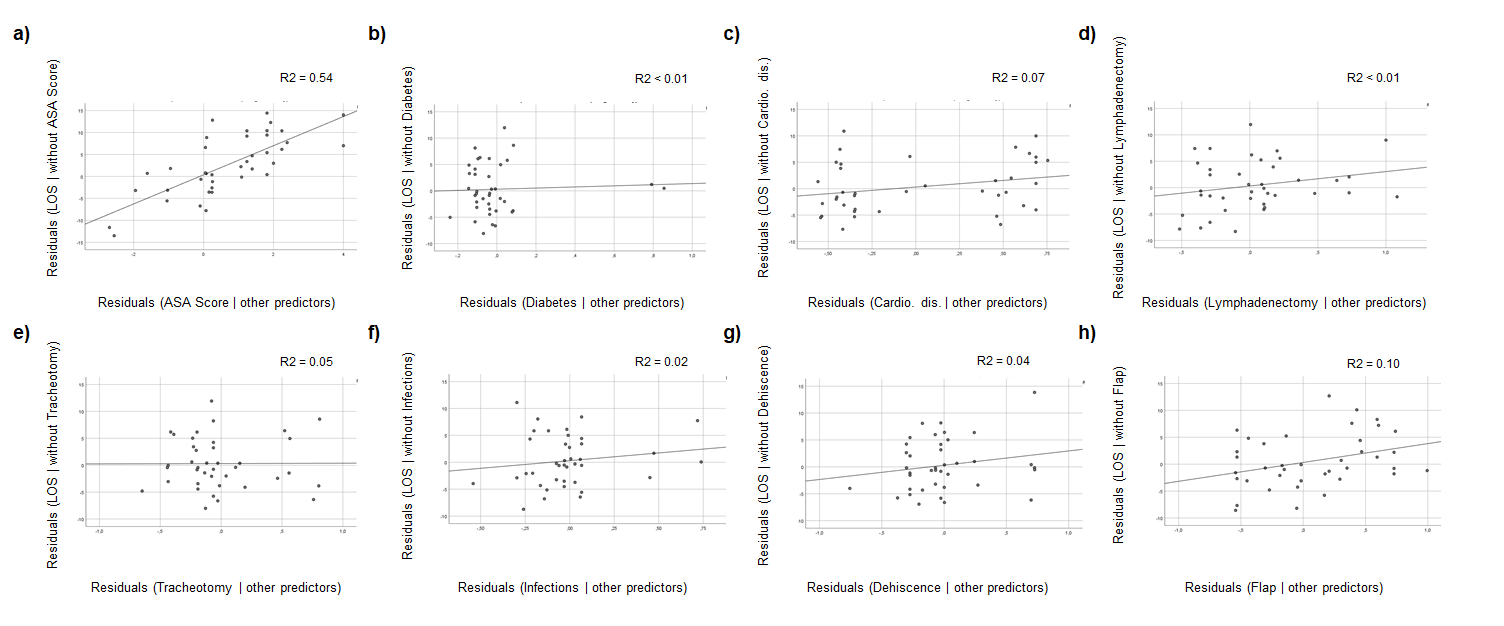


**Figure S2.** Partial regression plots for Cefamezyn plus Clyndamicin displayed on each predictor: **a)** ASA Score; **b)** Diabetes; **c)** Cardiovascular disease; **d)** Lymphadenectomy; **e)** Tracheotomy; **f)** Infections; **g)** Dehiscence; **h)** Flap. Determination coefficient R2 is also reported for each subplot.

The plots show a weak linear relationship between the LOS and the selected predictors. For both antibiotics, ASA Score has the highest R2.

### **1.2 INDEPENDENCE OF THE RESIDUALS**

The Durbin Watson test is used to check autocorrelation (Table S1). Values lower than 1 and higher than 3 indicate positive and negative autocorrelation respectively.

**Table S1.** Durbin Watson test.

| **Antibiotic** | **Durbin Watson test results** |
| --- | --- |
| Ceftriaxone | 1.258 |
| Cefamezyn plus Clyndamicin | 1.351 |

Results of the Durbin Watson test are in an acceptable range (higher than 1 and lower than 4), so we can assume no autocorrelation in the data, i.e. the values of the residuals of the model are independent.

### **1.3 COLLINEARITY**

In order to verify that all the predictors are independent each other’s (absence of collinearity), Tolerance and Variance Inflation Factors are calculated and reported in Table S2.

**Table S2.** Collinearity statistics.

| **Independent variables** | **Ceftriaxone** | | **Cefamezyn plus Clyndamicin** | |
| --- | --- | --- | --- | --- |
|  | **Tolerance** | **Variance Inflation Factors** | **Tolerance** | **Variance Inflation Factors** |
| ASA score | 0.243 | 4.121 | 0.326 | 3.068 |
| Oral hygiene | 0.390 | 2.564 | *n.a.* | *n.a.* |
| Diabetes | 0.711 | 1.407 | 0.823 | 1.214 |
| Cardiovascular disease | 0.427 | 2.345 | 0.519 | 1.926 |
| Lymphadenectomy | 0.736 | 1.358 | 0.458 | 2.184 |
| Flap | 0.701 | 1.427 | 0.356 | 2.809 |
| Tracheostomy | *n.a.* | *n.a.* | 0.487 | 2.055 |
| Infections | *n.a.* | *n.a.* | 0.595 | 1.681 |
| Dehiscence | *n.a.* | *n.a.* | 0.542 | 1.847 |

*n.a. = not applicable because the variable is not included in the model.*

Results are in the acceptable range, being the Tolerance higher than 0.2 and the Variance Inflation Factors lower than 10, for all the independent variables.

### **1.4 PRESENCE OF OUTLIERS**

The presence of influential cases biasing the model is checked by plotting the Cook’s distance and the Centered Leverage Value (Figure S3).


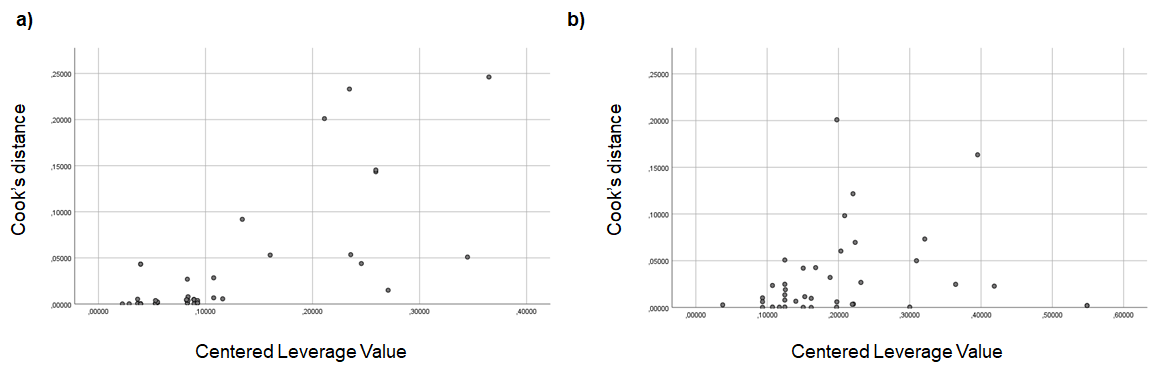


**Figure S3.** Cook’s distance is plotted vs the Centered Leverage Value for: **a)** Cefriaxone; **b)** Cefamezyn plus Clyndamicin.

In particular, the higher the Cook’s distance, the more influential the point is. We eliminated cases with Cook’s distance higher than 1 (3 outliers for Cefriaxone and 6 for Cefamezyn plus Clyndamicin). Therefore, graphs in Figure S3 show Cook’s distances after the removal of outliers. As a results, no points above the set threshold of 1 are identified.

### **1.5 NORMALITY DISTRIBUTION OF THE RESIDUALS**

The normality distribution of the residuals, is checked by looking at the Probability-Probability (Figure S4).


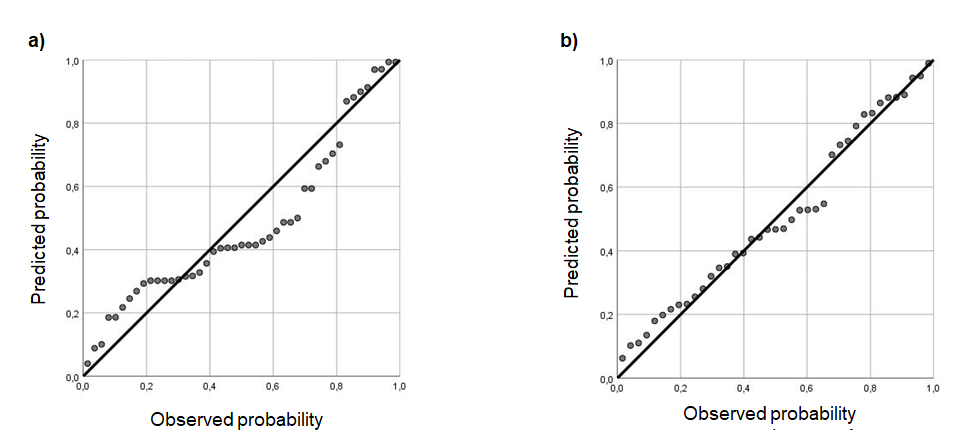


**Figure S4.** Probability-Probability plot for: **a)** Cefriaxone; **b)** Cefamezyn plus Clyndamicin.

No severe violation of the normality is present for Cefamezyn plus Clyndamicin while a slight deviation from the diagonal is present for Cefriaxone.

### **1.6 HOMOSCEDASTICITY OF THE DATA**

Homoscedasticity is checked by looking at the plot of the standardized residuals against the standardized predicted values obtained from the model (Figure S5).


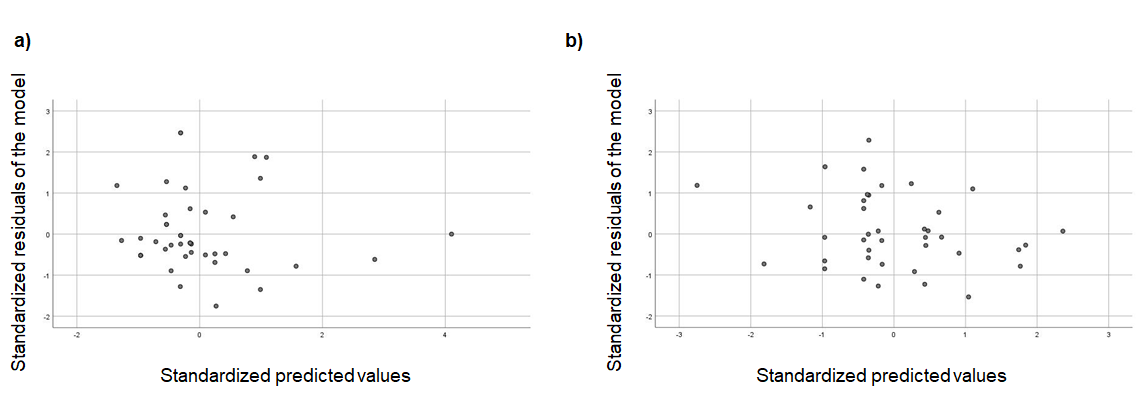


**Figure S5.** Standardized residuals vs standardized prediceted values for: **a)** Cefriaxone; **b)** Cefamezyn plus Clyndamicin.

Since the variation of the residuals is almost constant around the mean, no significant heteroscedasticity is detectable.
